# Knockout of Toll-Like Receptors 2 and 4 Prevents Renal Ischemia-Reperfusion-Induced Cardiac Hypertrophy in Mice

**DOI:** 10.1371/journal.pone.0139350

**Published:** 2015-10-08

**Authors:** Mayra Trentin-Sonoda, Rogério Cirino da Silva, Fernanda Vieira Kmit, Mariana Vieira Abrahão, Gustavo Monnerat Cahli, Guilherme Visconde Brasil, Humberto Muzi-Filho, Paulo André Silva, Fernanda Freire Tovar-Moll, Adalberto Vieyra, Emiliano Medei, Marcela Sorelli Carneiro-Ramos

**Affiliations:** 1 Centro de Ciências Naturais e Humanas, Universidade Federal do ABC, Santo André, Brazil; 2 Instituto de Biofísica Carlos Chagas Filho, Universidade Federal do Rio de Janeiro, Rio de Janeiro, Brazil; 3 Instituto Nacional de Ciência e Tecnologia de Biologia Estrutural e Bioimagem, Rio de Janeiro, Brazil; 4 Instituto D’Or de Pesquisa e Ensino, Rio de Janeiro, Brazil; 5 Instituto de Ciências Biomédicas, Universidade Federal do Rio de Janeiro, Rio de Janeiro, Brazil; Virginia Commonwealth University Medical center, UNITED STATES

## Abstract

We investigated whether the pathways linked to Toll-like receptors 2 and 4 (TLRs) are involved in renal ischemia-reperfusion (I/R)-induced cardiac hypertrophy. Wild type (WT) C57BL/6J, TLR2^-/-^ and TLR4^-/-^ mice were subjected to left kidney ischemia for 60 min followed by reperfusion for 5, 8, 12 and 15 days. Proton density magnetic resonance showed alterations in the injured kidney from WT mice, together with signs of parenchymal edema and higher levels of vimentin mRNA, accompanied by: (i) small, but significant, increase in serum urea after 24 h, (ii) 100% increase in serum creatinine at 24 h. A serum peak of inflammatory cytokines occurred after 5 days of reperfusion. Heart weight/body weight and heart weight/tibia length ratios increased after 12 and 15 days of reperfusion, respectively. Cardiac hypertrophy markers, B-type natriuretic peptide (BNP) and α-actin, left ventricle mass, cardiac wall thickness and myocyte width increased after 15 days of reperfusion, together with longer QTc and action potential duration. Cardiac TLRs, MyD88, HSP60 and HSP70 mRNA levels also increased. After 15 days of reperfusion, absence of TLRs prevented cardiac hypertrophy, as reflected by similar values of left ventricular cardiac mass and heart weight/body weight ratio compared to the transgenic Sham. Renal tissular injury also ameliorated in both knockout mice, as revealed by the comparison of their vimentin mRNA levels with those found in the WT on the same day after I/R. The I/R TLR2^-/-^ group had TNF-α, IFN-γ and IL-1β levels similar to the non-I/R group, whereas the TLR4^-/-^ group conserved the p-NF-κB/NF- κB ratio contrasting with that found in TLR2^-/-^. We conclude: (i) TLRs are involved in renal I/R-induced cardiac hypertrophy; (ii) absence of TLRs prevents I/R-induced cardiac hypertrophy, despite renal lesions seeming to evolve towards those of chronic disease; (iii) TLR2 and TLR4 selectively regulate the systemic inflammatory profile and NF- κB activation.

## Introduction

Cardiac hypertrophy (CH) is one of the most important causes of heart disease. Left ventricular hypertrophy, in particular, results in the development of cardiovascular disease (CVD) and heart failure, making them the most common causes of morbidity and mortality worldwide [1, 2]. Regarding the possible causes of CH, immunological alterations have been considered highly responsible [3, 4], placing the innate immune system as part of the phenomenon. This system acts on numerous tissues, with the factors involved including the toll-like receptors (TLRs), which have generally been described as recognizing pathogen-associated molecular patterns (PAMPs) [5]. TLRs cannot only recognize PAMPs, but damage-associated molecular patterns (DAMPs) [6, 7].

Among TLRs, the one most highly expressed in the heart is TLR4 [8]. TLR4 knockout mice develop less cardiac hypertrophy induced by pressure overload [9]. Most TLRs use an adaptor molecule, the protein product of myeloid differentiation primary gene 88 (MyD88), lack of which can change downstream signaling of TLR2 and TLR4, or even make it impossible. Ha *et al*. [10] showed that blocking MyD88 attenuates cardiac hypertrophy *in vivo*. Due to activation of the TLRs pathway, dependent or not of MyD88, nuclear factor kappa-B (NF-κB) becomes activated and translocates to the nucleus [11], where it promotes transcription of specific genes, including some related to the immune response, e.g. interferon-gamma (IFN-γ), interleukin-6 (IL-6) and tumor necrosis factor alpha (TNF-α), all mediators of inflammation [10–13], and strongly associated with the mechanism of cardiac hypertrophy [14–16]. These same molecules are also associated with acute and chronic renal disease [17, 18].

The incidence of kidney disease increases every year and is a major concern in many countries [19]. It is estimated that one in 10 American adults have some level of chronic kidney disease (CKD) [National Kidney and Urologic Diseases Information Clearinghouse], and individuals with CKD are more likely to develop cardiovascular disease [19]. Once kidney function is impaired and there is a deficit in glomerular filtration rate, several hemodynamic factors are altered, followed by toxins and other unwanted molecules starting to accumulate in the bloodstream [20]. Several cytokines and chemokines increase during kidney disease, consequently compromising the functioning of other organs.

We set out therefore to: (i) test the hypothesis that a unilateral renal I/R model can induce cardiac hypertrophy; and, if confirmed, (ii) assess whether TLR2 and TLR4 are involved in renal I/R-induced CH.

## Materials and Methods

### Animals and surgical procedures: the renal I/R protocol

All surgical procedures and protocols were performed in accordance with the Ethical Principles in Animal Research set forth by the Brazilian College of Animal Experimentation, with approval from the Biomedical Sciences Institute/University of São Paulo Ethics Committee for Animal Research (Book 20, Protocol 36, page 68). As in previous studies [21, 22], we used male wild type (WT) C57BL/6J mice, 5–8 weeks old (22–28 g), obtained from the University of São Paulo, Institute of Biomedical Sciences. TLR2 and TLR4 knockout mice (originally provided by Dr. Shizuo Akira, Osaka University, Osaka, Japan), all of them on the C57BL/6 background [6, 23], were kindly provided by Dr. Sergio Costa Oliveira (Federal University of Minas Gerais, Belo Horizonte, Brazil). Mice were given free access to standard mouse chow and water until the time of the experiment and were housed in a temperature- and light-controlled environment (24°C; 12/12 h-light/dark cycle). The protocol for renal I/R was that of Feitoza *et al*. [21]. The mice were anesthetized by i.p. injection of 116 mg/kg body weight ketamine (Agribands of Brazil Ltda, Paulínia, Brazil) and 11.5 mg/kg body weight xylazin (Agribands of Brazil Ltda). An abdominal incision gave access to the renal pedicle, and the organs were reflected outwards until the end of the occlusion procedure. The renal left pedicle was cleaned with a surgical forceps and occluded with a steel clamp (DL Micof, São Paulo, Brazil). During ischemia, mouse organs were kept moist with pre-warmed physiological saline. After 60 min occlusion, the steel clamp was removed and the animals allowed to recover prior to euthanasia. Sham-operated controls were subjected to surgical opening and closing in the abdominal region with gentle external manipulation of the vascular pedicle without occlusion.

After removal of the clamp, reperfusion was followed for 5, 8, 12 or 15 days in the I/R group. Except where otherwise indicated, the Sham group went for 15 days and were killed on day 15 with the last I/R group. Urea and creatinine were measured using commercial kits as given in the respective figure legends (Analisa, Belo Horizonte, Brazil or Labtest Diagnóstica, Lagoa Santa, Brazil). Vimentin levels, as a marker of renal injury, were measured by real time PCR (Stratagene® Mx3005P, Agilent Technologies, Santa Clara, CA). Animals under the anesthesia were killed by exsanguination after puncturing the inferior vena cava. To confirm the occurrence of acute I/R-induced renal lesions, urea and creatinine were measured in serum samples taken at 24, 48 and 72 h after I/R. Progress toward CKD was biochemically assessed by urea determinations from 5 to 15 days, as described below. For gene expression, protein content and cytokine dosage, tissues removed from mice were snap-frozen in liquid N_2_ and stored at -80°C until processed.

### Gene expression and protein content

Total RNA was extracted from heart and kidney using Trizol® (Invitrogen™/Life Technologies, Carlsbad, CA). Briefly, slices of hearts and kidneys were washed with saline and homogenized in Trizol®; chloroform was used as organic solvent. The aqueous phase was treated with isopropanol to precipitate the RNA, and the pellets were washed with an aqueous solution of ethanol 75% (v/v). RNA concentration and yield were assessed using NanoDrop Lite spectrophotometer (Thermo Scientific^©^, San Jose, CA). After reverse transcription, the final cDNA product was used to carry out real-time PCR (Stratagene® Mx3005P, Agilent Technologies) to quantify gene expression. mRNA levels for vimentin, TLR2, TLR4, MyD88, NF-κB p105, HSP60 and HSP70 were measured using the primers given in S1 Table. Western blot analyses were done as before [24], using primary anti-GAPDH (Santa Cruz Biotechnology Inc®, Santa Cruz, CA), anti-NF-κB p65 (Abcam, Cambridge, MA), anti-NF-κB phosphor S536 (Cell Signaling Technology, Boston, MA), followed by peroxidase-conjugated secondary antibodies (Cell Signaling Technology, and GE Healthcare, Little Chalfont, UK). Membranes were analyzed by the ECL system (Thermo Fisher Scientific®, Waltham, MA). Band densitometry used Image J software (Image J®) after film digitalization.

### Cytokine dosage

Blood was extracted and centrifuged at 3,000 rpm for 15 min using a clinical centrifuge to prepare serum. Samples were stored at -80°C pending experimental procedures. A Bioplex Assay Kit (Bio-Rad, Hercules, CA) was used to measure serum cytokine levels of TNF-α, IFN-γ and IL-1β in WT and knockout mice; the chemokine KC was measured in serum from WT mice as a biomarker of renal injury in the same way. Plates were read with a Luminex® 100/200 (Bio-Rad) system.

### Morphometric and histological examination

After euthanasia, the hearts were removed, weighed and analyzed morphometrically and histologically in the Sham and I/R group at selected times after surgical intervention (8, 12, 15 and 20 days). The aim was 2-fold: (i) measurement of the ventricular wall thickness; (ii) measurement of the left ventricular luminal volume. The hearts were weighed and measurements of 3 axes were obtained (length, width and thickness) using images taken with a Motic® camera, analyzed with Motic Images Plus 2.0 software (Xiamen, China). After removal of the atria and large vessels of the base, the left ventricle was transversely sectioned into 3 parts of 3 mm thickness between apex and base for stereological calculation of its luminal volume, according to the Cavalieri´s principle [25]. Images were recorded at different days to assess the evolution with time of ventricular wall thickness, cross-sectional luminal area and lumen ventricular volume.

For histological examination, cross-sectional slices at the mid-portion were removed and fixed in 10% neutral-buffered formalin (Sigma-Aldrich, St. Louis, MO) at room temperature for 48–72 h. The slices were dehydrated in an increasing alcohol series, immersed in benzene for 20 min, and embedded in paraffin. The samples were sectioned at 5 μm, 12 non-consecutive sections were collected, and these were stained with hematoxylin and eosin. Cardiomyocyte width was measured in 5 fields per slice from 3 mice in each group at a magnification of ×400 with a camera and Motic Image Plus 2.0 Software.

### ECG and action potential recording

Surface electrocardiograms were recorded from conscious mice. The QT, RR, QTc and QRS were analyzed. Action potentials from the left ventricle were recorded as previously described [26]. The parameters measured were action potential duration at 70% repolarization, action potential amplitude, maximal negative slope, triangulation and resting membrane potential.

### Assessment of left ventricular structure and function using echocardiography

*In vivo* transthoracic echocardiography used a high resolution imaging system (VEVO 770, VisualSonics, Toronto, Canada) equipped with a 30 MHz scan head (VisualSonics). Mice were placed in an induction chamber with a constant inflow of 5% isoflurane and 95% O_2_. When the mice were asleep, they were removed from the induction chamber, weighed and placed on a heated platform with electrocardiogram contact pads (THM 100, Indus Instruments, Houston, TX) and kept anesthetized using a nose-cone with 1–3% isoflurane and 9997% O_2_. Excess gases were evacuated passively using an activated charcoal absorption filter (Vapor Guard, Vet Equip, Pleasanton, CA). A rectal probe lubricated with gel was placed in the rectum and taped to the platform, and the temperature was maintained at 36.5–37.5°C. Depilatory cream applied to the chest was removed after 2 min for ultrasound gel to be applied. The ultrasound scan-head was placed in contact with the ultrasound gel for scanning over a 10 min period. B-mode and M-mode were obtained at the level of papillary muscle images. The temperature and heart rate were constantly monitored during scanning. Measurements were made offline using analytic software (VisualSonics). Electrocardiogram kilohertz-based visualization (EKV™) software analysis produced offline reconstruction for simulated 250–1000 Hz static and cine-loop images. Echocardiographic measurements included ventricular wall thickness and chamber size. EKV™ images in the modified parasternal short and long axes were used to measure left ventricular mass (LVM) using the area–length method:

$$\text{LVM }\left(\text{in g}\right)\;=\;1{.05\text{ g/cm}}^{3}´\{[\left(5/6\right)\text{ LV epicardial area}´\left(\text{LV long axis in diastole }+\text{ myocardial thickness}\right)]\;-\;[\left(5/6\right)\text{ LV endocardial area}´\left(\text{LV long axis in diastole}\right)]\}$$ (1)

where 1.05 g/cm^3^ is taken as the density of cardiac muscle. Modified parasternal long-axis EKV loops were also used to measure the ejection fraction (EF) by Simpson’s method (Simp). M-mode images were used to measure left ventricular (LV) chamber sizes and wall thicknesses. The percentage shortening fraction, reflecting left ventricular functioning through a diastolic dimension that is lost in systole, was calculated from M-mode measurements, using the leading edge to leading edge method according to the formula:

% Shortening Fraction (%SF) = 100´[LVID(d)] − [LVID(s)]/LVID(d) (2)

where LVID(d) is the left ventricular internal diameter in diastole and LVID(s) is the left ventricular internal diameter in systole.

The cardiac output [CO (calc)] was derived using the formula:

$$\text{CO }\left({\text{in cm}}^{3}\text{/min}\right)\;=\text{ SV }\left(\text{calc}\right)\times \left(\text{HR}\right)$$ (3)

where SV is the systolic volume and HR the heart rate.

### Image-guided injection of contrast agent and KCl

Image-guided intravenous injection (i.g.) involved a high resolution imaging system (VEVO 770, VisualSonics, equipped with a 30 MHz scan head RMV 770B) and anesthesia equipment for an inhaled mixture containing 1–3% isoflurane and 97–99% O_2_. The syringe was secured in a micromanipulator, and the needle and the RMV scan-head probe were aligned prior to injection so that the needle was at ~45° to the table surface. After alignment had been confirmed, the needle was retracted from the ultrasound field of view using the micromanipulator, and the mice were moved into position for echocardiographic visualization. Special care was taken to visualize the targeted vessel, ensuring that its position matched the previously aligned needle position. The needle was advanced with the micromanipulator under echo guidance through the vessel wall and the injection was performed.

### Magnetic resonance imaging

Magnetic resonance imaging (MRI) of the Sham and I/R groups was taken 15 days after renal surgery. For contrast-enhanced images, a 0.3 mmol/kg bolus of gadolinium-diethylenetriaminepenta-acetic acid (Gd-DTPA) was injected into the jugular vein. After 15 min, the animals were killed with a lethal injection of 1 ml 75% KCl at the same site as the contrast agent.

Images were acquired with a 7T magnetic resonance scanner (7T/400 horizontal Varian scanner, Agilent Technologies, Santa Clara, CA). A T1-weighted 3D gradient echo-imaging sequence (repetition time = 3.2 ms; echo time = 20 ms; flip angle = 30°; matrix: 96 × 96; field of view = 20 mm × 20 mm x 20 mm; slice thickness: 1 mm; 7–9 continuous slices, no gap; 6 averages) was used to investigate cardiac morphology. To investigate renal changes, abdominal images were taken as proton density sequences (repetition time = 10 ms; echo time = 2000 ms; matrix: 128 × 128; slice thickness: 1 mm; 15 continuous slices, no gap; 6 averages) in the axial (field of view = 26 mm × 26 mm) and coronal (field of view = 20 mm × 30 mm) planes. Prior to image analysis, datasets were anonymized and randomized across the groups. All the images for each dataset were visually inspected for artifacts. Data were processed with Osirix Software (www.osirix-viewer.com). Cardiac and renal morphology and imaging signal intensity were assessed by 2 experienced researchers.

### Statistics

Statistical analyses are based on GraphPad Prism 5 software (La Jolla, CA). Data are expressed as mean, SD or SEM, as indicated. Groups have been compared using one-way analysis of variance (ANOVA) followed by Bonferroni post-test or Student t-test, as indicated in the legends of figures and tables; *P* < 0.05 was taken as significant.

## Results

### Renal I/R induced renal dysfunction and exacerbated systemic inflammation in C57BL/6J wild type mice

**Fig 1:**
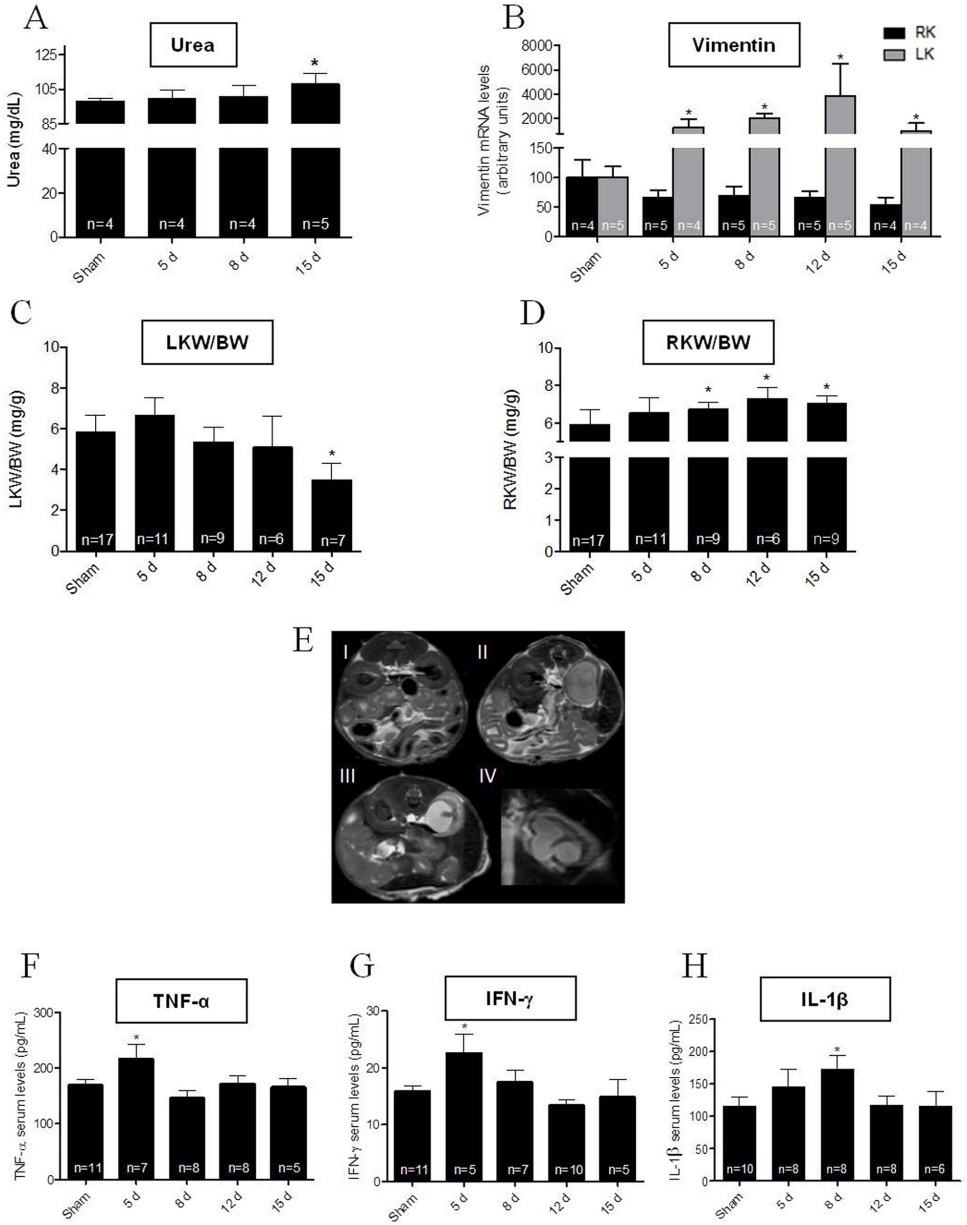
Characterization of renal ischemia reperfusion (I/R) model in C57BL/6J wild type mice. (A) Serum urea levels were measured with the Analisa kit in Sham mice (first column) and the I/R group at days 5, 8 and 15 after injury. (B) Vimentin mRNA levels in renal tissue (black bars, right kidney; gray bars, left kidney) were measured in the Sham (first column) and I/R groups at days 5, 8, 12 and 15 after injury. (C) Left kidney weight /body weight ratio (LKW/BW) measured in Sham mice and animals killed at the times after I/R indicated on the abscissa. (D) RKW/BW: the same for right kidney. (E) Proton density magnetic resonance renal images. I: axial plane view in a Sham mouse 15 days after surgical manipulation; II and III: axial planes of proton density magnetic resonance images from 2 different I/R mice 15 days after reperfusion; IV: coronal view of the abnormal kidney shown in III. (F to H) Serum levels of TNF-α, IFN-γ and IL-1β measured in the Sham group (0 day) and in mice after I/R at the days indicated on the abscissa. Bars data are mean ± SD. * *P* < 0.05 with respect to the corresponding Sham group (one way ANOVA followed by Bonferroni post-test for selected pairs; number of animals within the bars, where applied).

As a consequence of renal I/R, urea levels were 11% higher after 15 days of reperfusion, compared with those found in Sham mice (Fig 1A). This corresponds to a second phase of increase in serum urea after an early sustained increase over 24 h of I/R (S1 Fig panel A), along with a peak (~100% increase) in serum creatinine, which returned to near Sham values 24 h later (S1 Fig panel B). These observations indicate that an acute I/R process took place, evolving toward CKD with time following the initial injury. Analysis of the renal tissue injury marker, vimentin, over the following 15 days (Fig 1B) was also indicative of left kidney injury, along with the findings of a decrease by 40% in the mRNA levels of vimentin in the right kidney (black bars in Fig 1B), possibly reflecting recovery after surgery and vascular pedicle manipulation. There was a 40% decrease in the left kidney weight/body weight ratio 15 days after I/R (Fig 1C), whereas the opposite occurred in the right kidney, i.e. a small but significant and sustained increase of ~15% in the index about day 8 following injury compared to the Sham control (Fig 1D). There were also anatomical changes and signs of parenchymal edema in the left kidney at day 15, in contrast to the contralateral partner, data consistent with unilateral (left) kidney injury. While the axial plane of the proton density magnetic resonance image showed bilaterally normal renal morphology at 15 days in Sham animals (Fig 1E I), abnormal renal morphology was seen in the left kidney, including reduction of renal parenchymal thickness and increased image signal intensity (Fig 1E II and 1E III), abnormalities also evident in the coronal view (Fig 1E IV). Since a renal lesion is classically associated with an increase in systemic inflammation [27], serum cytokines were measured. Post-operation, the inflammatory marker TNF-α (at day 5), IFN-γ (day 5) and interleukin IL-1β (day 8) increased by 30, 40 and 35%, respectively, compared to the 15 day Sham control (Fig 1F to 1H). There was also a peak of the chemokine KC on day 5 after I/R: 86.5 ± 44.2 pg/mL (mean ± SD), which gradually returned to control values 15 days after reperfusion.

### Renal I/R induced cardiac hypertrophy

**Fig 2:**
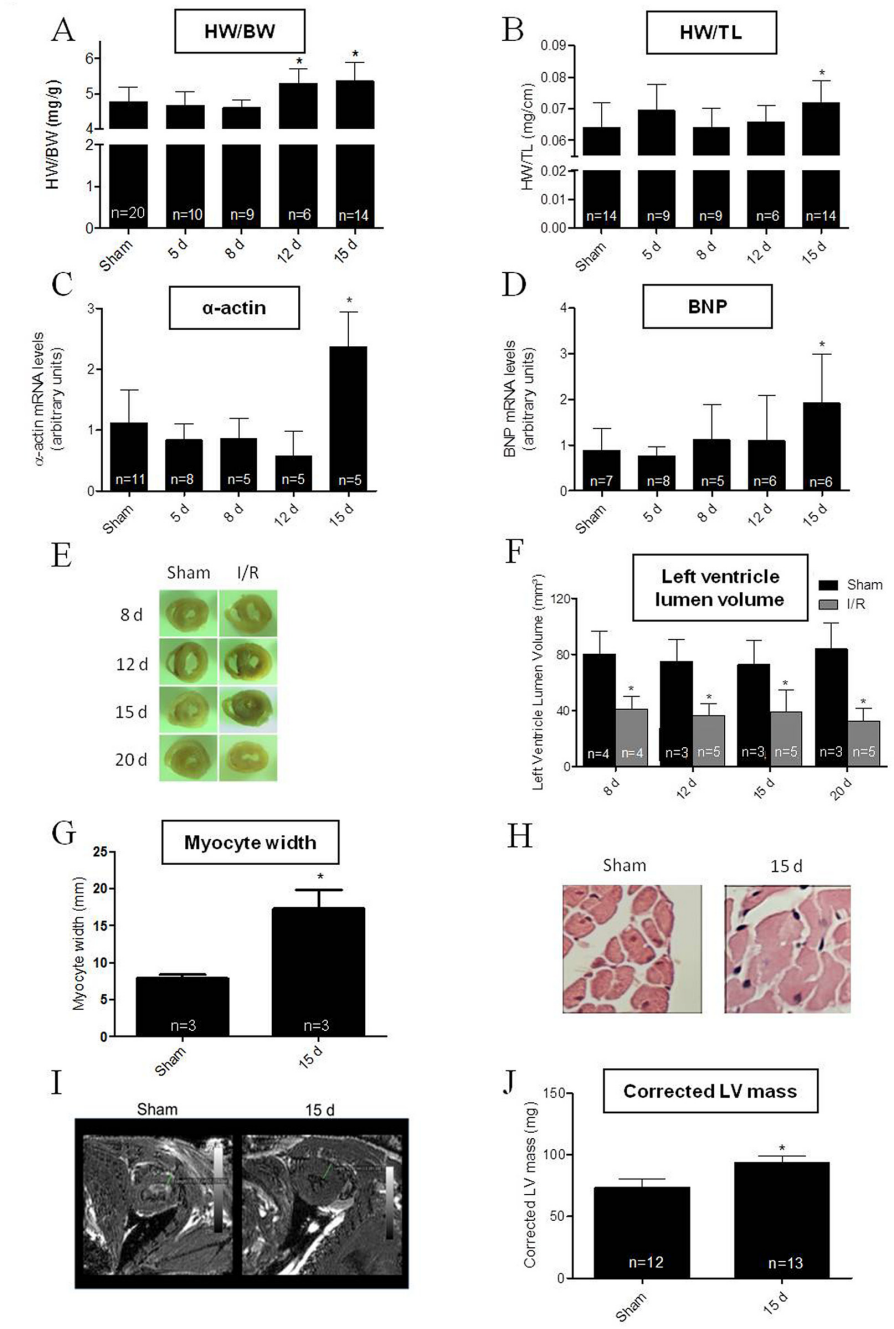
Cardiac hypertrophy induced by renal I/R in C57BL/6J wild type mice. (A) Heart weight/body weight (HW/BW) ratio was determined in Sham mice (15 days after surgical procedures) and the I/R group at the days after reperfusion indicated on the abscissa. (B) Heart weight/tibia length (HW/TL) ratio measured in the same groups and days. (C and D) Cardiac α-actin and BNP mRNA levels were assayed in hearts from Sham mice and at the given days after reperfusion in the I/R mice. In A to D, asterisks indicate significant differences (*P* < 0.05) with respect to the corresponding Sham group (one way ANOVA followed by Bonferroni post-test for selected pairs). Data are mean ± SD. (E) Representative images from medium slices of heart showing the luminal area of the left ventricle in Sham and I/R mice at the days after surgery indicated on the left-hand side of the figure. (F) Left ventricle lumen volumes were determined in Sham (black bars) and I/R mice (gray bars) at the days after intervention as indicated on the abscissa. Data are mean ± SD. * *P* < 0.05 with respect to the corresponding day-matched Sham group (one-way ANOVA followed by Bonferroni post-test for selected pairs). (G) Representative hematoxylin and eosin stained ventricular slices obtained from Sham and I/R mice after 15 days after of the intervention; magnification ×400. (H) Graphical representation of myocyte width obtained using hematoxylin and eosin stained ventricular slices form the number of Sham and I/R mice indicated within the bars. * *P* < 0.05 with respect to Sham (Student’s t-test). (I) Axial planes of a T1-weighed 3D gradient echo ventricular imaging sequences from Sham and I/R mice 15 days after intervention. (H) Graphical representation of corrected left ventricle (LV) mass obtained from echocardiography in Sham and I/R. Data are mean ± SD. * *P* < 0.05 with respect to Sham (Student’s t-test).

Intervention in the left kidney disturbed some cardiac parameters. The data strongly suggest cardiac hypertrophy developed by 12–15 days of renal reperfusion. The heart weight/body weight ratio increased by 15% compared to the 15 day Sham controls (Fig 2A), an index matching a comparable increase in the heart weight/tibia length ratio (Fig 2B). Levels of α-actin and B-type natriuretic peptide (BNP) mRNAs showed a burst of >100% in the I/R group after 15 days compared to the Sham controls (Fig 2C and 2D), reflecting a well-established biochemical cardiac lesion being coincident with a left renal lesion. Sectioning of heart tissue showed an early reduction in the left ventricular luminal area in I/R mice (Fig 2E), accompanied by a significant decrease in left ventricular lumen volume (Fig 2F). Another important indicator of cardiac hypertrophy was increased cardiomyocyte width at 15 days of reperfusion (Fig 2G and 2H). Magnetic resonance images show a thicker ventricular wall (Fig 2I), suggesting concentric hypertrophy in agreement with the pronounced decrease in left ventricle volume (Fig 2F). This ensemble of findings were corroborated by echocardiography, that showed an increase in both left ventricle mass and corrected left ventricular mass concomitantly (Fig 2J, Table 1).

**Table 1:** Echocardiogram parameters 15 days after I/R.

	% SF	% EF	LV Mass (AW)	Corrected LV Mass	CO
Sham (n = 12)	49.1 ± 2.6	57.3 ± 2.8	94.9 ± 5.5	73.3 ± 6.7	22.3 ± 3.3
I/R (n = 13)	51.8 ± 3.1	59.1 ± 3.3	113.0 ± 4.1*	93.3 ± 5.7*	25.4 ± 2.9

SF: Shortening fraction; EF: Ejection fraction; LV: Left ventricle; AW: Anterior wall; CO: Cardiac output. Echocardiogram parameters were calculated according to Eqs 1–3 (see text). Data expressed as mean ± SEM.

*P < 0.05 with respect to the corresponding Sham (Student t-test).

The echocardiographic parameters were calculated using Eqs 1–3 (see text).

**Fig 3:**
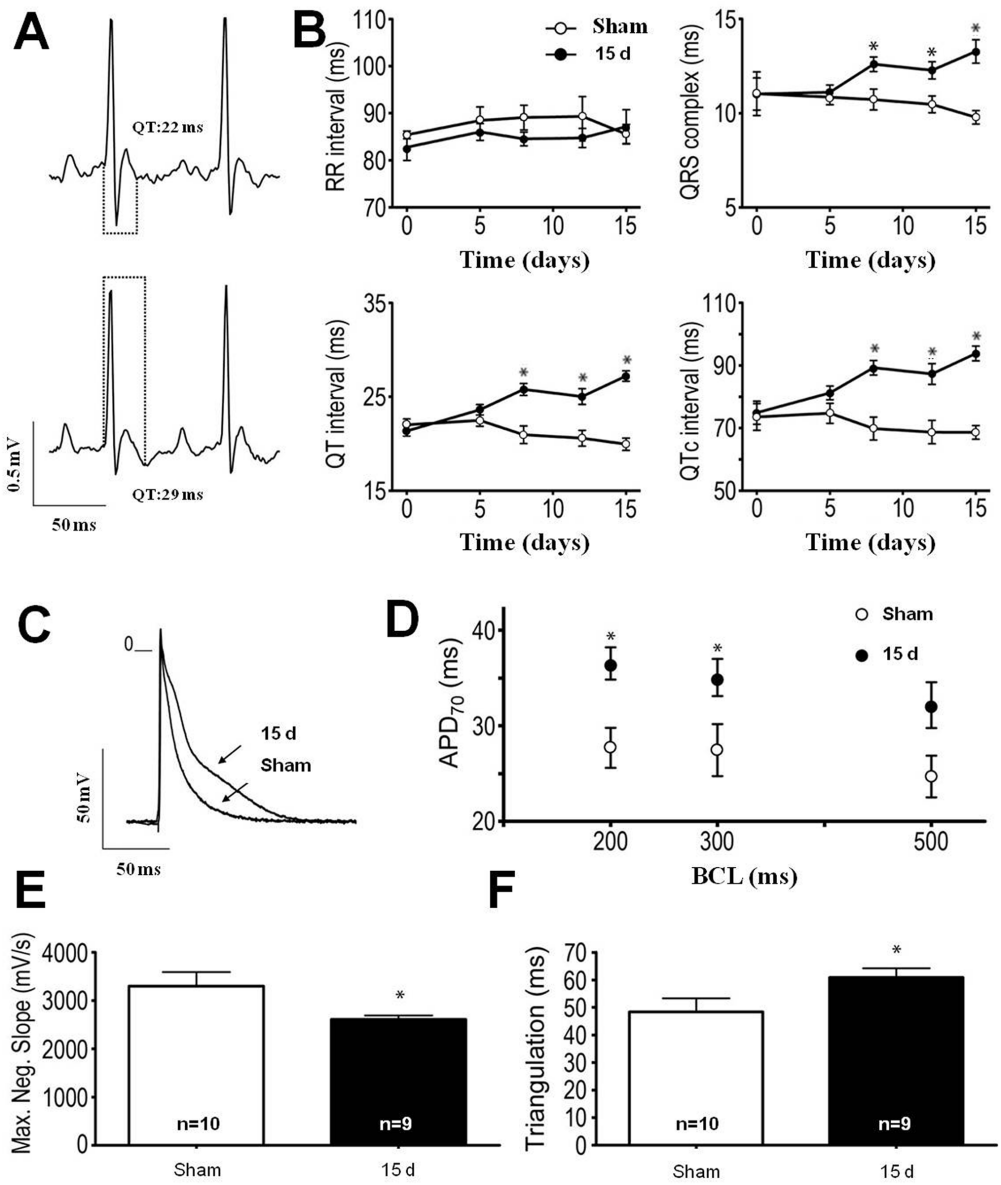
Renal I/R-induced cardiac electrical disturbances in C57BL/6J wild type mice. (A) Representative traces of electrocardiograms from Sham (upper) and I/R mice (bottom) 15 days after intervention. Dots show the differences in QT duration. (B) Temporal follow-up of RR, QRS, QT and QTc at 0, 5, 8, 12 and 15 days after pedicle manipulation (Sham, n = 10) or reperfusion release (I/R mice, n = 9). Data are mean ± SEM. * *P* < 0.05 with respect to the corresponding day-matched Sham group (one way ANOVA followed by Bonferroni post-test for selected pairs). (C) Representative action potential traces from left ventricle endocardial tissue recorded from a Sham mouse and an I/R mouse 15 days after intervention. (D) This graph summarizes the duration of action potential at 70% repolarization (APD_70_) under different basic cycle lengths (BCL) of stimulation (200, 300 and 500 ms). * *P* < 0.05 with respect to the corresponding BCL-matched 15 days Sham group (one-way ANOVA followed by Bonferroni post-test for selected pairs). (E and F) These bar graphs summarize the maximal negative slope of repolarization (E) and triangulation duration (F) found in Sham and 15 days I/R mice. Data are mean ± SEM. *P < 0.05 with respect to Sham (Student t-test). Recordings from 5–7 hearts; 7–9 cells per heart.

Fig 3A gives a representative prolonged QT interval (29 ms) in an ECG recorded from a kidney I/R mouse 15 days after injury compared with a day-matched Sham control mouse (22 ms). Interestingly, although most of the morphological changes appeared at 12–15 days after reperfusion, electrocardiography detected changes earlier; at day 8 after reperfusion, prolonged QRS, QT and QTc were found, with no modification in the RR interval (Fig 3B; Table 2). There was also an increase in action potential duration (a representative trace is given in Fig 3C) and in action potential duration at 70% of repolarization (APD_70_) under different basic cycle lengths (BCL) of stimulation (Fig 3D; Table 3). Maximal negative slope of repolarization was decreased in the 15 days group, and triangulation was higher in the I/R group than in the Sham controls (Fig 3E and 3F). Interestingly, while electrical function was impaired at days 12–15 after I/R, mechanical ventricular function seemed to be preserved, ultrasonography showing no change in shortening fraction, ejection fraction or cardiac output, despite an overall increase in left ventricular mass (Table 1).

**Table 2:** Cardiac electrical profile "in vivo".

Groups										
	0 d		5 d		8 d		12 d		15 d	
Parameter	Sham	I/R	Sham	I/R	Sham	I/R	Sham	I/R	Sham	I/R
RR (ms)	88.5 ± 2.8	86.0 ± 1.8	89.1 ± 2.8	86.0 ± 1.7	89.1 ± 2.6	84.6 ± 1.4	89.3 ± 4.2	84.8 ± 2.4	85.6 ± 2.0	87.1 ± 3.6
QRS (ms)	11.0 ± 1.1	11.0 ± 0.3	10.9 ± 0.8	11.1 ± 0.4	10.7 ± 0.3	12.6 ± 0.4*	10.5 ± 0.3	12.3 ± 0.4*	9.8 ± 0.5	13.3 ± 0.6*
QT (ms)	22.0 ± 0.6	21.4 ± 0.6	22.5 ± 0.5	23.6 ± 0.8	21.0 ± 0.6	25.8 ± 0.8*	20.6 ± 0.5	25.0 ± 0.7*	20.0 ± 0.9	27.2 ± 0.5*
QTc (ms)	73.6 ± 4.2	74.9 ± 2.3	74.8 ± 3.6	81.3 ± 3.7	69.9 ± 3.2	89.3 ± 3.3*	68.7 ± 2.2	87.3 ± 3.3*	68.7 ± 3.6	93.8 ± 2.3*

The results are expressed in mean ± SEM.

* *P* < 0.05 vs. the day-matched Sham parameter (Student t-test).

Sham n = 10, I/R n = 9.

**Table 3:** Cardiac electrical profile "in vitro" 15 days after I/R.

Variables	Sham	I/R
RMP (mV)	-68.3 ± 1.4	-67.8 ± 1.2
APA (mV)	93.6 ± 3.3	93.5 ± 1.4
Max.Neg.Slope (mV/s)	3303.0 ± 287.0	2612.0 ± 84.4*
Triangulation (ms)	48.4 ± 4.9	60.9 ± 3.3*
APD_70_−500 (ms)	24.7 ± 2.1	32.2 ± 2.3
APD_70_−300 (ms)	27.5 ± 2.7	35.1 ± 1.9*
APD_70_−200 (ms)	27.7 ± 2.0	36.5 ± 1.6*

RMP: Resting membrane potential; APA: Action potential amplitude; Max.Neg.Slope: Maximal negative slope; APD: Action potential duration at different BCL (500, 300 and 200 ms). The results are expressed in mean ± SEM.

* *P* < 0.05 vs. the corresponding Sham.

Recordings from 5–7 hearts, 7–9 cells per heart.

### Cardiac Toll-like receptors, MyD88, NF-κB and HSPs increase in I/R-induced cardiac hypertrophy

**Fig 4:**
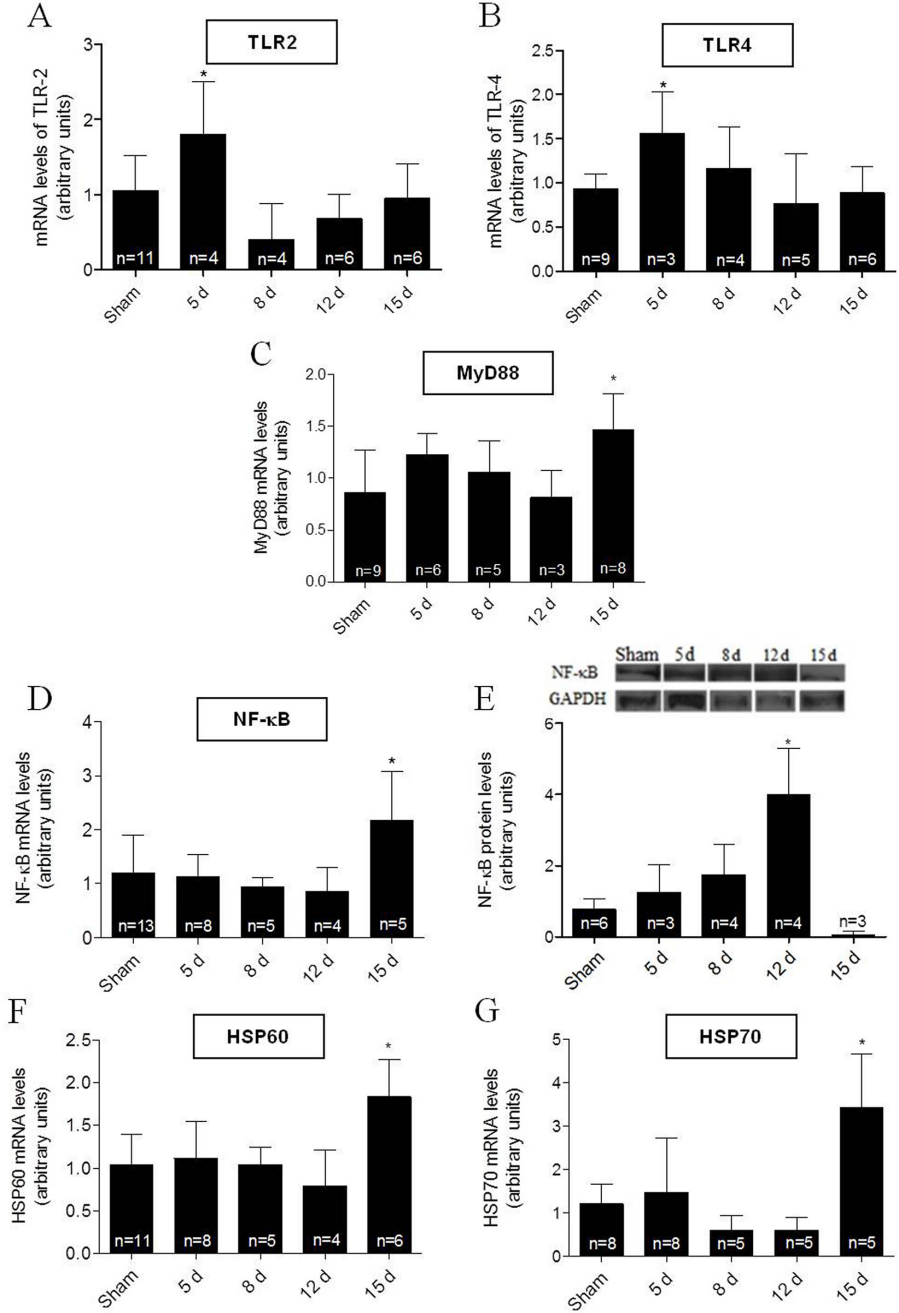
Cardiac TLR2 and 4 pathway after renal I/R in C57BL/6J wild type mice. Measurements were carried out in heart tissues at the times after I/R given on the abscissae. In all experiments, the Sham group corresponds to assays carried out using mice 15 days after intervention. (A to D) mRNA expression levels of TLR2, TLR4, MyD88 and NF-κB p105. (E) NF-κB p105 protein abundance; GAPDH: control for protein loading. (F and G) mRNA expression levels of HSP60 and HSP70, respectively. Data are mean ± SD; n: indicated within the bars. *P < 0.05 compared to the corresponding Sham group (one-way ANOVA followed by Bonferroni post-test for selected pairs).

Participation of TLR signaling in the basic mechanism of cardiac hypertrophy development has been reported [9–11]. Therefore, to assess their possible participation in renal I/R-induced cardiac hypertrophy, mRNA expression of key molecules involved in this pathway was explored. A peak in the expression of both cardiac TLR2 and TLR4 mRNAs occurred at day 5 after I/R (Fig 4A and 4B), which was earlier than the peak expression of both the downstream MyD88 and NF-κB p105 mRNAs that came at 15 days of reperfusion (Fig 4C and 4D). Protein levels of NF-κB were increased at 12 days (Fig 4E). Since the crucial activation of both TLR2 and TLR4 by HSPs is already known [28], we assessed cardiac mRNA expression of HSP60 and HSP70. Peak mRNA expression of both HSPs occurred at day 15 compared to the 15 day Sham group (Fig 4F and 4G).

### Knockout of TLR2 and TLR4 prevents renal I/R-induced cardiac hypertrophy

**Fig 5:**
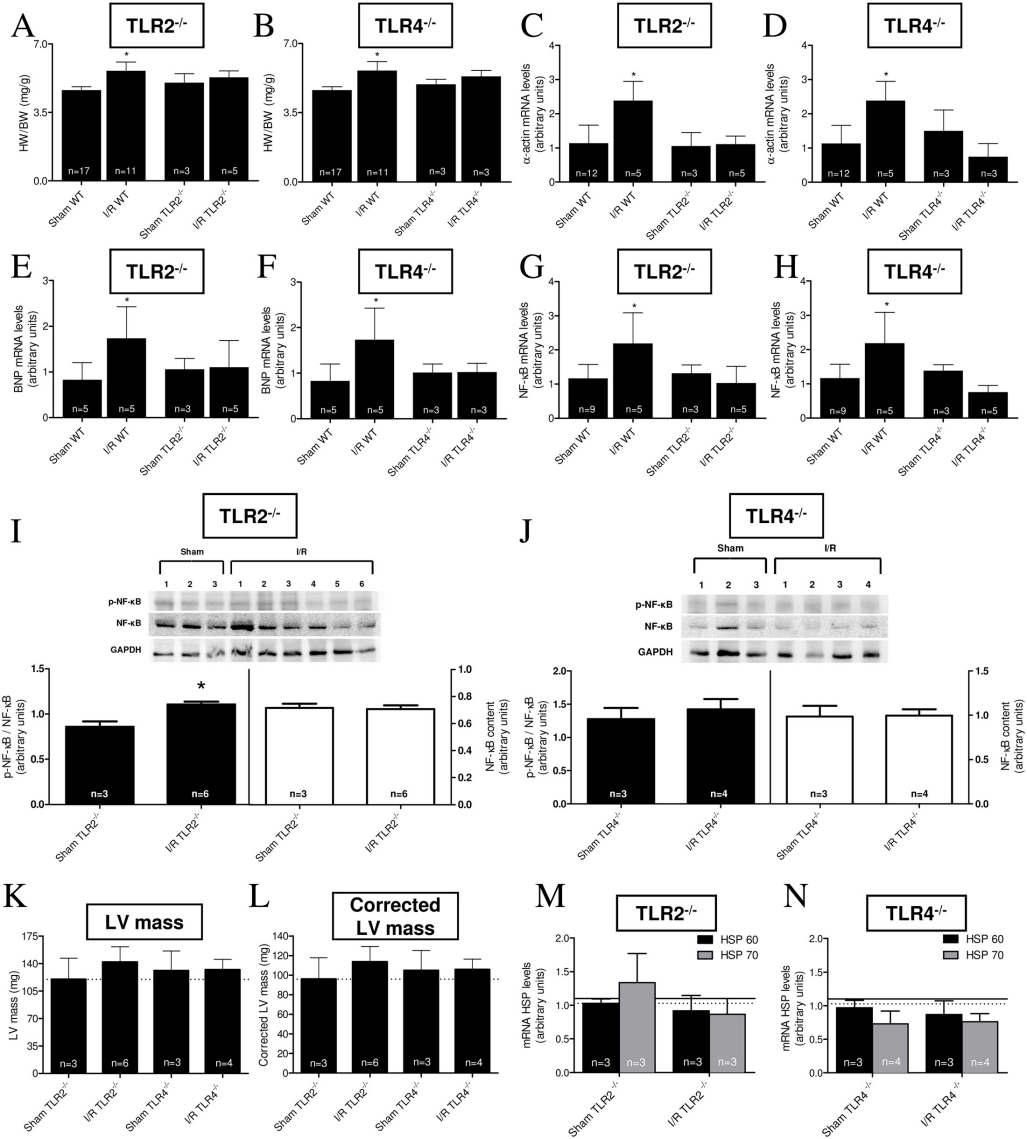
Assays in TLR2 and 4 knockouts strongly suggest the participation of TLR2 and 4 in I/R-induced cardiac hypertrophy. (A to H) HW/BW ratios, α-actin mRNA levels, BNP mRNA levels and NF-κB p105 mRNA levels assayed in TLR2^-/-^ or TLR4^-/-^ mice (indicated at the top of their respective panels). Each determination was carried out in Sham mice (15 days after intervention) or I/R mice 15 days after injury. The values for each parameter were compared with those obtained with WT mice, as indicated on the corresponding abscissae. The number of determinations is indicated within the bars. *P < 0.05 with respect to the corresponding Sham group (one-way ANOVA followed by Bonferroni post-test for selected pairs). (I and J) p-NF-κB/NF-κB ratio and total unphosphorylated NF-κB content in cardiac TLR2^-/-^ and TLR4^-/-^ mice in Sham and I/R conditions after 15 days; n: indicated within the bars and the gels, where each lane was loaded with cardiac homogenate from different mice. GAPDH was the housekeeping protein for control of loading. Black bars: p-NF-κB/NF-κB ratio; empty bars: total unphosphorylated NF-κB content. *P < 0.05 with respect to the corresponding Sham (Student’s t-test). (K and L) Left ventricle (LV) mass and corrected LV mass values determined in Sham and I/R TLR2^-/-^ or TLR4^-/-^ mice, 15 days after intervention. Dashed lines represent WT mice values. Data are mean ± SD; the number of determinations is indicated within the bars. No statistical differences were found when the I/R group from each knockout was compared to its corresponding Sham group (Student’s t-test). (M and N) HSP60 (black bars) and HSP70 (gray bars) mRNA levels in TLR2^-/-^ and TLR4^-/-^ (Sham and 15 days I/R mice). Solid line and dashed lines represent WT values for HSP60 and HSP70, respectively. Data are mean ± SD; the number of determinations is indicated within the bars. No statistical differences were found when the I/R group for each protein in TLR2^-/-^ or TLR4^-/-^ was compared to the corresponding knockout Sham (Student t-test).

Knockout mice helped to determine whether TLR2 or TLR4 were key participants in I/R-induced cardiac hypertrophy. In both TLR2^-/-^ and TLR4^-/-^, an increase in HW/BW ratio evident 15 days after I/R was prevented compared to transgenic Sham mice (Fig 5A and 5B). Regarding the molecular markers, α-actin and BNP, their mRNA levels increased by ~100% after I/R in WT mice, whereas both types of knockout mice were totally insensitive to the injury regarding these markers (Fig 5C to 5F). The same pattern in both TLR2^-/-^ and TLR4^-/-^ was found with respect to the downstream mRNA expression of NF-κB p105 (Fig 5G and 5H). Western blotting showed that the p-NF-κB/NF-κB ratio increased in the TLR2^-/-^ I/R group, while TLR4^-/-^ I/R was unchanged compared to TLR4^-/-^ Sham mice (Fig 5I and 5J). LV mass assessed by ultrasonography was not significantly different in either TLR2^-/-^ or TLR4^-/-^ I/R mice compared with the transgenic Sham mice (Fig 5K and 5L). Since, as already mentioned, HSPs are natural TLR activators and their mRNAs are increased in hearts from WT mice after 15 days of reperfusion, we assessed the HSP 60 and 70 mRNA levels after the same period in both groups of transgenic mice. Fig 5M and 5N clearly demonstrate that there was no increment of cardiac expression of either of the HSPs mRNAs after I/R in both knockout mice compared to their corresponding Sham controls.

**Fig 6:**
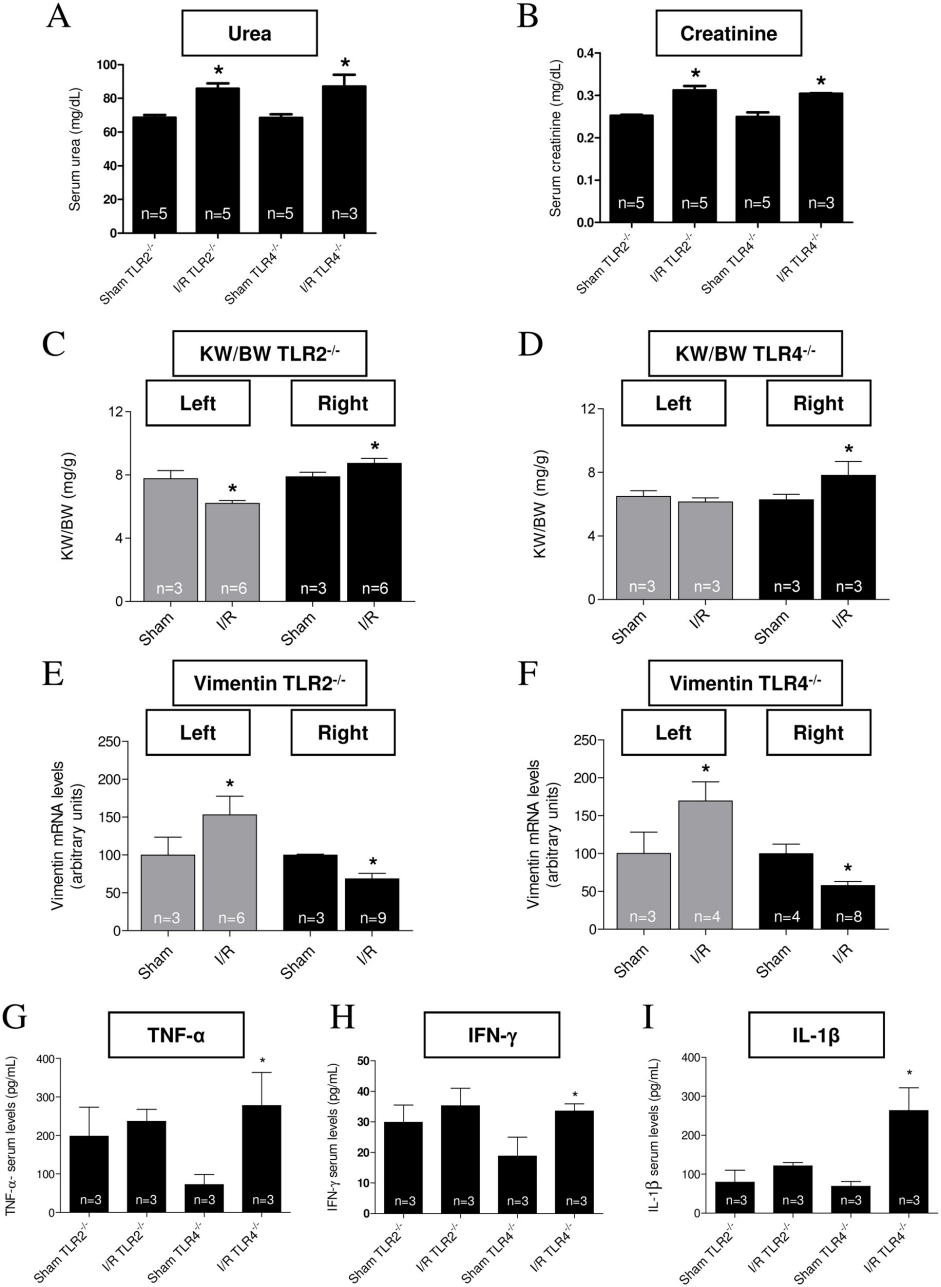
Renal status in TLR2^-/-^ and TLR4^-/-^ 15 days after I/R. Data are mean ± SD; number of animals within the bars. (A and B) Serum urea and creatinine levels in TLR2^-/-^ and TLR4^-/-^ from I/R mice measured using the Analisa kit, and compared to those from the corresponding Sham (15 days after intervention), as indicated on the abscissae. * *P* <0.05 with respect to the Sham group (one-way ANOVA followed by Bonferroni post-test for selected pairs). (C and D) Kidney weight/body weight ratio (KW/BW) in TLR2^-/-^ and TLR4^-/-^ (Sham and I/R mice 15 days after intervention). Gray bars: left kidney; black bars: right kidney. (E and F) Renal vimentin mRNA levels in TLR2^-/-^ and TLR4^-/-^ (Sham and I/R mice 15 days after chirurgical intervention). Gray bars: left kidney; black bars: right kidney. In C to F: * *P* < 0.05 with respect to the corresponding Sham kidney (one way ANOVA followed by Bonferroni post-test for selected pairs). (G to I) TNF-α, IFN-γ and IL-1β serum levels from TLR2^-/-^ and TLR4^-/-^ mice (Sham and I/R, as shown on the abscissae, 15 days after intervention). *P < 0.05 when the I/R group was compared with the corresponding Sham (one-way ANOVA followed by Bonferroni post-test for selected pairs).

To assess whether the absence of TLRs in renal tissue could prevent unilateral kidney injury, renal function/structure was compared in Sham and I/R TLRs knockout mice (TLR2^-/-^ and TLR4^-/-^). After 15 days of reperfusion, both plasma urea and creatinine concentrations were significantly increased in I/R groups compared to the day-matched Sham controls (Fig 6A and 6B). The decrease in the left renal index KW/BW in the I/R TLR2^-/-^ group compared to Sham control (Fig 6C, gray columns) is once again indicative of the persistence of renal lesion at 15 days, as seen in WT mice (Fig 1C). Conversely, the index increased in the contralateral kidney (Fig 6C, black bars). In the knockout TLR4^-/-^, the left kidney index remained unmodified 15 days after I/R (Fig 6D, gray bars), but the left side injury clearly increased in the right kidney after I/R (Fig 6D, black bars). With respect to the lesion biomarker, vimentin, this was higher in the left kidney of both TLR2^-/-^ (Fig 6E, gray bars) and TLR4^-/-^ mice (Fig 6F, gray bars) after I/R. Conversely, vimentin mRNA expression decreased in the right kidney of both I/R TLR2^-/-^ and TLR4^-/-^ mice (Fig 6E and 6F, black bars). TLR2^-/-^ mice, but not TLR4^-/-^ mice, showed improvement of their systemic inflammatory status, reflected by similar concentrations of TNF-α, IFN-γ and IL-1β after I/R, which had greatly increased in the TLR4^-/-^ mice after the injury (Fig 6G to 6I).

Vimentin mRNA levels increased by >800% in WT left kidney (Fig 1B), whereas—at the same time—the values were 55% and 70% in TLR2^-/-^ and TLR4^-/-^, respectively (Fig 6E and 6F). Conversely, the former had an 11% increase in serum urea with respect to Sham (Fig 1A), in contrast with the higher increase in TLR2^-/-^ and TLR4^-/-^ (25 and 27%, respectively).

## Discussion

Renal I/R promotes acute renal lesions, which become evident within 72 h of injury [21, 22], characterized by an exacerbated inflammatory state [29, 30]. Using the same model as in [21], we show that acute I/R lesions—clearly confirmed by a huge peak of serum creatinine 24 h after reperfusion together with a gradual rise in urea (S1 Fig)–progressively leads to impairment of renal structure and function. After cytokine peaks at day 5 (TNF-α and IFN-γ) and day 8 (IL-1β) (Fig 1F to 1H), and a gradual increase of the inflammatory marker, vimentin, in left renal tissue (Fig 1B), a status of CKD is firmly established at day 15 after injury, confirmed by biochemical (Fig 1A), anatomical (Fig 1D) and magnetic resonance scanning imaging (Fig 1E). The cytokine KC, considered to be a circulating biomarker of renal ischemic injury [31], is of particular relevance for the confirmation of the occurrence of a real I/R process. Together these findings allow us to hypothesize that the ischemic protocol exacerbates a systemic inflammatory condition, generating increases in systemic cytokines [27] and activation of TLRs, impairing cardiac structures and functions as one of its major consequences.

Driven by the hypothesis that the primary kidney acute I/R injury can culminate with a cardiorenal syndrome [32, 33] with several degrees of cardiac structural and electrical dysfunctions, we examined morphological, molecular and functional parameters that would reflect an increase in cardiac mass. Typically, cardiac hypertrophy is characterized by elevated molecular markers, e.g. α-actin and BNP [34–36]. Heart weight/body weight and heart weight/tibia length ratios are also useful indirect indicators of cardiac hypertrophy [35, 36], and these parameters increased after 12–15 days of reperfusion (Fig 2A to 2D). Other parameters classically related to cardiac hypertrophy were also measure, including left ventricular lumen area, left ventricular wall thickness, cardiomyocyte width, and corrected left ventricular mass. After 12–15 days of reperfusion, all the data indicated the development of cardiac hypertrophy (Fig 2E to 2H).

Cardiac electrical profiles “in vivo” and “in vitro” are also in line with the morpho-structural alterations described above. The longer QRS, QT and QTc seen after 8 days of reperfusion and sustained till day 15 of reperfusion (Fig 3A and 3B; Table 2), are consistent with data demonstrating the association of these electrocardiographic changes with cardiac hypertrophy [34, 36]. Of special relevance is the longer QT interval that, together with the “in vivo” electrophysiology alterations, is considered a predictor of arrhythmias and sudden death [37, 38]. The slower maximal negative slope of repolarization, with longer APD duration and triangulation (Fig 3C to 3F) shows anomalous behavior in terms of cardiac ionic currents as a consequence of renal I/R. Interestingly, electrical changes preceded mechanical dysfunction assessed by ECHO: shortening fraction, ejection fraction and cardiac output were unchanged at 15 days after I/R (Table 1). In several pathological situations, electrical disturbances precede mechanical dysfunction and failure [39, 40], and also seems to be the case for the renal I/R-induced cardiac anomalies. Overall, cardiac morpho-structural and electrical modifications point to the predominant pathophysiological mechanism of a CKD cardiomyopathy, i.e. cardiac myocyte dysfunction [33].

The process of cardiac hypertrophic impairment is usually associated with increases in inflammatory molecules [27], as found in this study (Fig 1F to 1H). Others have found that inflammatory mediators can act locally or migrate through blood stream to reach other organs [13, 21, 22, 41, 42]. This might have happened after an intense inflammatory response in the left kidney, as confirmed by the progressive increase in vimentin (Fig 1B) with its impact in heart tissue. Interestingly, early blood cytokine peaks coincide with early and transient cardiac peaks (~80% higher than in the corresponding Sham groups) of both TLR2 and 4 mRNAs expression (Fig 4A and 4B), suggesting that these receptors mediated the cardiac hypertrophic response after cytokine release from an I/R-damaged left kidney scenario. This view is supported by the finding that cardiac hypertrophy is prevented in TLR4-deficient mice [9], and that TLRs are crucial inducers of cardiac hypertrophy [42–45] and cardiomyocyte apoptosis [46].

Looking downstream at the TLRs signaling route, there were increased cardiac MyD88 and NF-κB mRNA levels, as well as upregulation of NF-κB protein (Fig 4C to 4E). Triggering the TLRs pathway led to activation of the branch that involves the adaptor protein MyD88, amongst others [10–13, 28], and culminates with translocation of NF-κB to the nucleus [11]. This process may be crucial in the hypertrophic response of cardiomyocytes [28, 47–49]. Prevention of the I/R-induced cardiac hypertrophy in either TLR2^-/-^ or TLR4^-/-^ transgenic mice (5A and B) gives further support to the idea that these receptors act centrally in the signaling mechanisms of renal I/R-induced cardiac hypertrophy and inflammation. That a secondary inflammatory cardiac response after renal I/R sees NF-κB as a key player is supported by the fact that this transcription factor binds to target DNA sequences, thereby stimulating the expression of genes, including those coding for TNF-α, IL-6 and other inflammatory cytokines [10–13].

A noteworthy point is the late (and simultaneous) increase in mRNA levels of the intracellular chaperones, the heat shock proteins (HSPs; Fig 4F and 4G), which could contribute and amplify the TLRs-mediated signaling messages. Extracellular HSPs have already been shown to play a key role in cardiomyocyte hypertrophy and apoptosis mediated by TLR2 [28]. It may be that these proteins, acting from extracellular space, help to amplify the intracellular cardiac cascade involved in the hypertrophic response upon TLRs activation, as seen in several disease models [50, 51]. However, the possibility that the increased HSPs mRNA levels are more like a protective response against the cell stress represented by increased pro-inflammatory cytokines after renal I/R has to be considered, since HSPs have dual functions depending on their location [52]. Whatever the cause and relevance of this increase, Sham and WT levels of the HSPs mRNAs are maintained in the heart of the 2 knockout mice used, despite the induction of renal I/R (Fig 5M and 5N); this gives more support to the idea that TLRs and HSPs interact in the pathways culminating with I/R-induced cardiac hypertrophy and dysfunction.

Although several morphological and biochemical parameters (Fig 5A to 5F), as also NF-κB mRNA levels (Fig 5G and 5H), indicate that either TLR2 or TLR4 gene deletion are beneficial in preventing cardiac damage associated with renal I/R, an important difference in terms of mechanisms may become more evident when the abundance of the protein NF-κB and its phosphorylation status are taken together with the response to I/R (Fig 5I and 5J). The abundance of this transcription factor in heart tissue remains unmodified 15 days after I/R in both knockout mice, but the phosphorylation status in response to I/R—and possibly activation—of the protein seems to be dependent of TLR4, but not to the activation of TLR2. The p-NF-κB/NF-κB ratio increases after I/R, but only in the TLR2^-/-^ mice. This the question arises: how can we conciliate these observations with those showing that activation of TLR4 and participation of the adaptor MyD88 and p-NF-κB are required for cardiomyocytes survival [53]?. The answer may depend on the 2 different and opposite cytokines involved in these processes—beneficial IL-10 on survival and, among others, the pro-inflammatory TNF-α (Fig 1) in damage induced by I/R. Different network wiring and cross-signaling in the TLR4 pathway might help explain this apparent paradox.

In the light of cardiac changes discussed, we should also consider the renal structural and functional status in TLR2^-/-^ and TLR4^-/-^ mice 15 days after I/R. Since the preservation of cardiac-renal axis integrity is vital in all physiological scenarios, it could be hypothesized that prevention or attenuation of renal lesions after I/R injury contributes to prevent I/R-induced cardiac hypertrophy. This hypothesis is supported by several studies showing that TLR2^-/-^and TLR4^-/-^ and knockout mice seem to be protected against renal metabolic and vascular injuries [54–56]. In our case, despite renal lesions still existing after 15 days in knockout mice, they seem to be distinct from that encountered in WT animals, as shown by the huge differences in vimentin mRNA levels. The modest increase (~50%) encountered in TLR2^-/-^ and TLR4^-/-^ compared to Sham (Fig 6E and 6F) is clearly contrasting with the >800% rise in WT 15 days after I/R, and indicative of a beneficial influence of TLRs deletion in kidney tissue. Since vimentin is a marker of cells undergoing an epithelial → mesenchymal transition [57], TLR2^-/-^ and TLR4^-/-^ deletion clearly ameliorates lesions at renal interstitial level. On the other hand, their high urea and creatinine (Fig 6A and 6B) suggest the persistence of lesions at a glomerular level, coexisting with macroscopic left kidney atrophy in I/R TLR2^-/-^, but not in TLR4^-/-^ mice (Fig 6C and 6D, gray bars), a difference possibly linked to the selective influences of these receptors on NF-κB phosphorylation, as already discussed. I/R-induced right kidney mass compensation was also evident in both types of knockout mice (Fig 6C and 6D, black bars), and also point to a benefit of TLRs deletion. Since this increase was also seen in WT mice (Fig 1D), the process clearly occurs when at least one TLR is present. The mechanism of the right kidney compensation after left renal I/R seems to underpin—at least in part—the involvement of vimentin expression, which decreases in the contralateral kidney (Fig 6E and 6F, compare gray and black bars). These results suggest (i) some specific mediators are involved that have not previously been described, and (ii) indicate that the limitations of the study demand more careful functional and structural analyses to measure differences at kidney level in WT and knockout mice.

Finally, differences in the circulating cytokines in TLR2^-/-^ and TLR4^-/-^ mice have to be considered. To get a better understanding of how TLRs knockout avoid I/R-induced cardiac hypertrophy will require the study of the systemic inflammatory profile. In both TLR2^-/-^ and TLR4^-/-^ knockout mice, the systemic inflammatory state is attenuated in a diabetic rodent model [58, 59]. We have now demonstrated that TLR2^-/-^, but *not* TLR4^-/-^ mice, can maintain high serum levels of inflammatory-related cytokines (Fig 6G to 6I), which is data consistent with another study [60] showing that, in a doxorubicin-induced delayed cardiomyopathy model, TLR2^-/-^ mice have a significantly suppressed inflammatory state, whereas TLR4^-/-^ mice maintain an inflammatory profile similar to WT mice.

In conclusion, evidence is presented that cardiac TLR2 and TLR4 signaling pathways may be key in renal I/R-induced cardiac hypertrophy and electrical dysfunction through mechanisms that involve MyD88 and NF-κB. The different effects of these receptors on NF-κB phosphorylation, possibly related with the diversity of systemic inflammatory changes they induce, indicate that the role of these receptors can be selectively modulated.

## Supporting Information

S1 Fig Early increase in serum urea and creatinine concentrations after unilateral (left) I/R. Urea and creatinine were determined (Labtest Diagnóstica kit) in serum from Sham mice 72 h after the surgical intervention, and in I/R mice at the times indicated on the abscissa. Data are mean ± SD; number of mice within the bars. * *P* < 0.05 when compared to the corresponding Sham (one way ANOVA followed by Bonferroni post-test for selected pairs).
(PDF)

S1 Table List of primers for qRT-PCR experiments. (PDF)
