# Supplementary material for: Knockout of Toll-Like Receptors 2 and 4 Prevents Renal Ischemia-Reperfusion-Induced Cardiac Hypertrophy in Mice
Source: PLoS One. 2015 Oct 8;10(10):e0139350. doi: 10.1371/journal.pone.0139350 (PMC4598103; doi:10.1371/journal.pone.0139350)
Supplement: S1 Fig — Urea and creatinine were determined (Labtest Diagnóstica kit) in serum from Sham mice 72 h after the surgical intervention, and in I/R mice at the times indicated on the abscissa. Data are mean ± SD; number of mice within the bars. * P < 0.05 when compared to the corresponding Sham (one way ANOVA followed by Bonferroni post-test for selected pairs). (PDF) [file pone.0139350.s001.pdf]

S1 Fig.

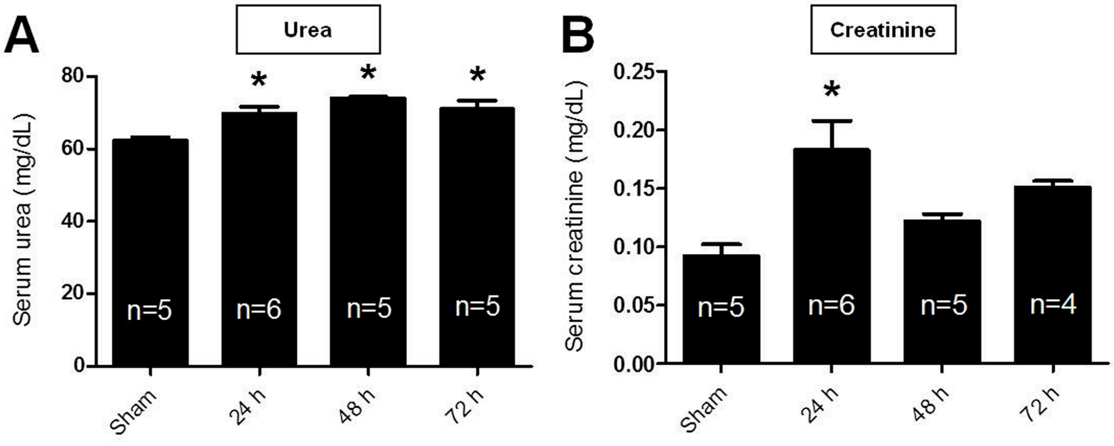

S1 Fig. Early increase in serum urea and creatinine concentrations after unilateral (left) I/R. Urea and creatinine were determined (Labtest Diagnóstica kit) in serum from Sham mice 72 h after the surgical intervention, and in I/R mice at the times indicated on the abscissa. Data are mean ± SD; number of mice within the bars. * $P < 0.05$ when compared to the corresponding Sham (one way ANOVA followed by Bonferroni post-test for selected pairs).
