# Supplementary material for: Knockout of Toll-Like Receptors 2 and 4 Prevents Renal Ischemia-Reperfusion-Induced Cardiac Hypertrophy in Mice
Source: PLoS One. 2015 Oct 8;10(10):e0139350. doi: 10.1371/journal.pone.0139350 (PMC4598103; doi:10.1371/journal.pone.0139350)
Supplement: S1 Table — (PDF) [file pone.0139350.s002.pdf]

S1 Table. List of primers for qRT-PCR experiments.

Genes	Forward	Reverse
Vimentin	5'-AGATCGATGTGGACGTTTCC-3'	5'-TCCGGTACTCGTTTGACTCC-3'
TLR2	5'-TGGGCAGTCTTGAACATTTG-3'	5'-GAAGTCAGCCCAGCAAAATC-3'
TLR4	5'-CAGCAAAGTCCCTGATGACA-3'	5'-TGCCTTGTCTTCAATTGTTTCA-3'
MyD88	5'-TGTCTCCAGGTGTCCAACAG-3'	5'-ATCTTCAGGGCAGGGACAA-3'
NF-κB p105	5'-CTACGGAACTGGGCAAATGT-3'	5'-CACACATAGCGGAATCGAAA-3'
HSP60	5'-GCCAAAGGGAAGAACTGTGA-3'	5'-CGTTTGTGTTATTGGCAACG-3'
HSP70	5'-CTGAACCCGCAGAACACC-3'	5'-TCTCGCCCTTGTAGTTCACC-3'
α-actin	5'-GGCAAGATGAGAGTGCACAA-3'	5'-CGGAGAATGATGGTCCAGAT-3'
BNP	5'-CAGCTCTTGAAGGACCAAGG-3'	5'-AGACCCAGGCAGAGTCAGAA-3'
